# Postsynaptic Target Specific Synaptic Dysfunctions in the CA3 Area of BACE1 Knockout Mice

**DOI:** 10.1371/journal.pone.0092279

**Published:** 2014-03-17

**Authors:** Hui Wang, Andrea Megill, Philip C. Wong, Alfredo Kirkwood, Hey-Kyoung Lee

**Affiliations:** 1 Department of Neuroscience, Mind/Brain Institute, Johns Hopkins University, Baltimore, Maryland, United States of America; 2 Department of Biology, University of Maryland, College Park, Maryland, United States of America; 3 Department of Pathology and Neuroscience, Johns Hopkins University School of Medicine, Baltimore, Maryland, United States of America; Torrey Pines Institute for Molecular Studies, United States of America

## Abstract

Beta-amyloid precursor protein cleaving enzyme 1 (BACE1), a major neuronal β-secretase critical for the formation of β-amyloid (Aβ) peptide, is considered one of the key therapeutic targets that can prevent the progression of Alzheimer’s disease (AD). Although a complete ablation of *BACE1* gene prevents Aβ formation, we previously reported that BACE1 knockouts (KOs) display presynaptic deficits, especially at the mossy fiber (MF) to CA3 synapses. Whether the defect is specific to certain inputs or postsynaptic targets in CA3 is unknown. To determine this, we performed whole-cell recording from pyramidal cells (PYR) and the stratum lucidum (SL) interneurons in the CA3, both of which receive excitatory MF terminals with high levels of BACE1 expression. BACE1 KOs displayed an enhancement of paired-pulse facilitation at the MF inputs to CA3 PYRs without changes at the MF inputs to SL interneurons, which suggests postsynaptic target specific regulation. The synaptic dysfunction in CA3 PYRs was not restricted to excitatory synapses, as seen by an increase in the paired-pulse ratio of evoked inhibitory postsynaptic currents from SL to CA3 PYRs. In addition to the changes in evoked synaptic transmission, BACE1 KOs displayed a reduction in the frequency of miniature excitatory and inhibitory postsynaptic currents (mEPSCs and mIPSCs) in CA3 PYRs without alteration in mEPSCs recorded from SL interneurons. This suggests that the impairment may be more global across diverse inputs to CA3 PYRs. Our results indicate that the synaptic dysfunctions seen in BACE1 KOs are specific to the postsynaptic target, the CA3 PYRs, independent of the input type.

## Introduction

Alzheimer’s disease (AD) is one of the most prevalent forms of senile dementia, but currently there is no effective treatment that can stop the progression of the disease [1]. A prevalent hypothesis on the etiology of the disease is the amyloid cascade hypothesis, which states that over-production of Aβ peptide initiates the pathogenesis of AD [1]–[3]. Aβ is produced by a sequential cleavage of amyloid precursor proteins (APPs) by β- and γ-secretases [1]. Because γ-secretase also has other physiological functions critical for normal cell development [4], [5], β-secretase inhibition has surfaced as a more attractive therapeutic option [1], [6]–[8]. However, many recent studies, including our own, have shown that although BACE1 knockouts (KOs) lack Aβ peptides [9] and show no gross anatomical or functional abnormalities [10], [11], they display specific synaptic dysfunctions in the CA1 and CA3 regions of the hippocampus [12]–[14]. In particular, BACE1 KOs showed presynaptic dysfunctions at the mossy fiber (MF) to CA3 synapses, which is one of the major loci of BACE1 expression in the brain [12]. By pinpointing the presynaptic dysfunction of BACE1 KOs to the level of presynaptic Ca^2+^ signaling [13], we were able to rescue the phenotype by activation of α7-nicotinic acetylcholine receptors (nAChRs) [15].

The CA3 pyramidal neurons not only receive robust excitatory inputs from MFs, but also receive strong feedforward inhibition from interneurons (INTs) within the stratum lucidum of CA3, which are also activated by MFs [16]. It is known that dentate granule cells mainly produce inhibition of CA3 pyramidal neurons via this feedforward circuit [16], [17]. Recent studies suggest that this feedforward inhibition controls the output of CA3 PYRs [18] and confers precision to memory encoding [19]. The stratum lucidum subfield of the CA3 is highly enriched in BACE1 protein [12]; hence it is pertinent to understand how knocking out BACE1 influences excitatory and/or inhibitory synapses in this feedforward inhibitory circuit. Many neuronal functions depend on a critical balance between excitatory and inhibitory circuits, thus understanding the impact of BACE1 inhibition on each synapse-type present in a circuitry is critical. Furthermore, understanding how lacking BACE1 activity affects specific synapses will aid in the development of effective methods to overcome the synaptic deficits and potentially benefit the therapeutics of AD. Therefore, in the current study, we examined the changes in circuit function in the CA3 area of hippocampus by performing whole-cell recording of excitatory synaptic transmission in pyramidal cells (PYRs) and the stratum lucidum inhibitory interneurons (SL-INTs), both of which receive MF inputs, as well as inhibitory inputs from SL to CA3 PYRs to specifically locate the synapses affected by losing BACE1 activity. We report here that BACE1 KOs display synaptic dysfunctions at both excitatory and inhibitory inputs to CA3 PYRs without changes in excitatory inputs to SL-INTs.

## Materials and Methods

### Animals

All mice used (BACE1+/+, +/−, and −/−; C57BL6 background) were derived from heterozygous breeders (+/−) as described previously [12]. The Institutional Animal Care and Use Committees of both University of Maryland and Johns Hopkins University approved all procedures involving animals.

### Acute Hippocampus Slices Preparation

Acute hippocampal slices were prepared from 16–28 days old BACE1 knockout (KO), heterozygous (HET) or wildtype (WT) mice as described previously [12], [13], [15]. Briefly, each mouse was euthanized by decapitation following overdose of isoflurane. Hippocampi were rapidly removed and sectioned into either 300-μm (for whole-cell recording) or 400-μm (for field potential recording) slices on a vibratome (Vibratome 3000, Ted Pella Inc.) using ice-cold dissection buffer (in mM: 212.7 sucrose, 2.6 KCl, 1.23 NaH_2_PO_4_, 26 NaHCO_3_, 10 dextrose, 3 MgCl_2_, and 1 CaCl_2_) saturated with 5% CO_2_/95% O_2_. The slices were transferred to a submersion-type holding chamber containing artificial cerebrospinal fluid (ACSF, in mM: 124 NaCl, 5 KCl, 1.25 NaH_2_PO_4_, 26 NaHCO_3_, 10 dextrose, 1.5 MgCl_2_, and 2.5 CaCl_2_, saturated with a mixture of 5% CO_2_/95% O_2_) and recovered for at least 1 hour at room temperature before being used for all experiments.

### Field Potential Recording from Mossy Fiber Pathway in CA3 Area

Recordings were done in a submersion-type chamber perfused with ACSF (29.5°C–30.5°C, 2 ml/min). Synaptic responses were evoked through glass bipolar stimulating electrodes placed in the dentate granule cell layer to activate MFs with half-maximal stimulation intensity (0.2 ms pulse duration at 0.067 Hz), and recorded extracellularly in the stratum lucidum of CA3. Paired-pulse facilitation (PPF) was measured at 25, 50, 100, 200, 400, 1000, and 2000 ms interstimulus intervals (ISIs). To induce mfLTP, three trains of 100 Hz (1 sec) stimuli were given at 20 sec intervals. All experiments were done in the presence of 100 μM D,L-2-amino-5-phosphonovaleric acid (D,L-APV) (Sigma-Aldrich) to isolate the presynaptic NMDAR-independent mossy fiber long-term potentiation (mfLTP) [20]. At the end of each experiment, 1 μM (2S,2′R,3′R)-2-(2′,3′-dicarboxycyclopropyl) glycine (DCG-IV) (Tocris Bioscience) was added, and blockade ≥80% were taken to be MF inputs. Field potential slopes were measured, and data are expressed as mean ± standard error of mean.

### Whole-cell Recording of Evoked Excitatory (eEPSCs) and Inhibitory (eIPSCs) Postsynaptic Currents

The slices were visualized by an upright microscope (Nikon E600FN) equipped with infra-red oblique illumination. CA3 PYRs and INTs located within the stratum lucidum of CA3 were initially identified on the basis of somata shape and position within the CA3 subfield, and patched by a whole-cell patch pipette (tip resistance, 3–5 MΩ). For eEPSC recordings, 20 μM bicuculline and 100 μM D,L-APV were added to ACSF to isolate AMPAR-mediated responses, and the recording pipette was filled with internal solution containing (in mM): 120 CH_3_O_3_SCs, 5 MgCl_2_, 8 NaCl, 1 EGTA, 10 HEPES, 0.001 QX-314, 0.5 Na_3_GTP, and 2 MgATP (pH 7.2–7.3, 280–290 mOsm). To record GABA_A_R-mediated eIPSCs, 10 μM NBQX (Sigma-Aldrich) and 100 μM D,L-APV were added to the ACSF, and eIPSCs were recorded with recording pipette filled with (in mM): 140 CsCl, 0.2 CaCl_2_, 8 NaCl, 2 EGTA, 10 HEPES, 0.5 Na_3_GTP, and 4 MgATP (pH 7.2, 275–285 mOsm), which allows recording of eIPSCs as inward currents at −70 mV holding potential. Biocytin (Sigma-Aldrich, 1 mg/ml) was routinely added to the recording electrode solution to allow post-hoc morphological identification of the recorded cells. Recordings were made at 30°C at a holding potential of −70 mV. Additionally, 150 nM CNQX (Tocris Bioscience) was added to prevent polysynaptic responses for eEPSCs in CA3 PYRs. For eEPSCs in INTs, CNQX was not used, as INTs were reported to show less recurrent excitation [21]. A double-barrel glass electrode filled with ACSF as stimulation electrode was placed in the dentate granule cell layer to activate the MF inputs. Minimum-intensity stimulation (15–30 μA intensity) with pulse duration of 0.2 ms (at 0.1 Hz) was used to induce monosynaptic responses. DCG-IV was applied at the end of the recordings to verify that eEPSCs were elicited by MF inputs. 20–30 waveforms were used to produce the average eEPSCs and eIPSCs, and the amplitude, latency, rise time, and decay time of the averaged trace was calculated by Igor Pro software (WaveMetrics). Paired pulse ratio was measured at 50 ms ISI. Only the cells and recording conditions that met the following criteria were studied: no obvious polysynaptic waveforms, R_input_≥125 MΩ, R_series_≤25 MΩ, and less than 15% change in R_series_ during the course of the experiment.

### Whole-cell Recording of Miniature Synaptic Events

To record AMPAR-mediated miniature excitatory postsynaptic currents (mEPSCs), the same intracellular solution as eEPSCs was used in the recording pipette, and 1 μM TTX (Tocris Bioscience), 20 μM bicuculline (Enzo Life Science) and 100 μM D,L-APV were added to the ACSF (29.5°C–30.5°C, 2 ml/min). For mIPSC in CA3 PYRs, the recording pipette was filled with (in mM): 140 CsCl, 8 KCl, 10 EGTA, 10 HEPES, and 0.01 QX-314 (pH 7.3, 275–285 mOsm), which allows recording of mIPSCs as inward current at negative holding potential, and 1 μM TTX, 100 μM D,L-APV, and 10 μM NBQX were added to the ACSF to isolate GABA_A_R-mediated currents. Both mEPSCs and mIPSCs were recorded at a holding potential of −70 mV using an Axopatch 700B amplifier (Molecular Devices), digitized at 10 kHz by a data acquisition board (National Instruments), and acquired using custom-made Igor Pro software (WaveMetrics). Acquired mEPSCs and mIPSCs were analyzed using the Mini Analysis program (Synaptosoft). The threshold for detecting mEPSCs was set at three times the root mean square (RMS) noise. mEPSCs with >3 ms rise time (measured between 10 and 90% of amplitude) and mIPSCs with >5 ms rise time (10 and 90% of amplitude) were excluded from analysis due to potential dendritic filtering. Average mEPSC and mIPSC amplitude and frequency were calculated and compared between two genotypes using unpaired Student’s t test as noted in text. For mIPSC frequency calculation, 350 to 500 consecutive events from each experiment were considered, but highly superimposed events constituting “bursts” (more than two events, interevent interval <10 ms) were excluded from the measurement of amplitudes (300 non-burst events from each cell were used for average mIPSC amplitude calculations). The decay time constant was calculated using the average of 150–200 well-isolated events. Only the cells with R_input_≥150 MΩ and R_series_≤25 MΩ were studied, and cells were discarded if R_input_ or R_series_ changed >15%.

### Immunohistochemistry and Confocal Imaging

Hippocampal slices with biocytin filled neurons were placed in 4% paraformaldehyde in 0.1 M PB (0.02 M NaH_2_PO_4_ and 0.08 M Na_2_HPO_4_) overnight at 4°C. After rinsed in 0.1 M PB (2×10 min), the slices were permeabilized in 2% Triton X-100 (TX-100)/0.1 M PB for 1 hour. They were then incubated in 1% TX-100/0.1 M PB solution with 1 μg/ml avidin-AF488 (Molecular Probes) overnight at 4°C. The slices were then rinsed in 0.1 M PB (2×10 min), and incubated in 300 nM DAPI/0.1 M PB solution for 1 minute. After washed in 0.1 M PB for 10 min, they were mounted on glass slides, and air-dried. The slides were coverslipped with Prolong™ mounting solution (Molecular Probes) and sealed with nail polish. The stained slices were imaged using a Leica SP5X confocal microscope with a 63× oil immersion objective lens. A UV laser was used to excite DAPI signal, and a 488-nm wavelength laser was used for biocytin staining. The CA3 subfield with biocytin filled neurons was imaged through the z-axis at 0.5 μm steps with x/y/z resolution of 0.24/0.24/0.50 μm/pixel.

## Results

### Young BACE1 KOs show Similar Synaptic Dysfunctions at Mossy Fiber to CA3 Synapses as Adults

**Figure 1:**
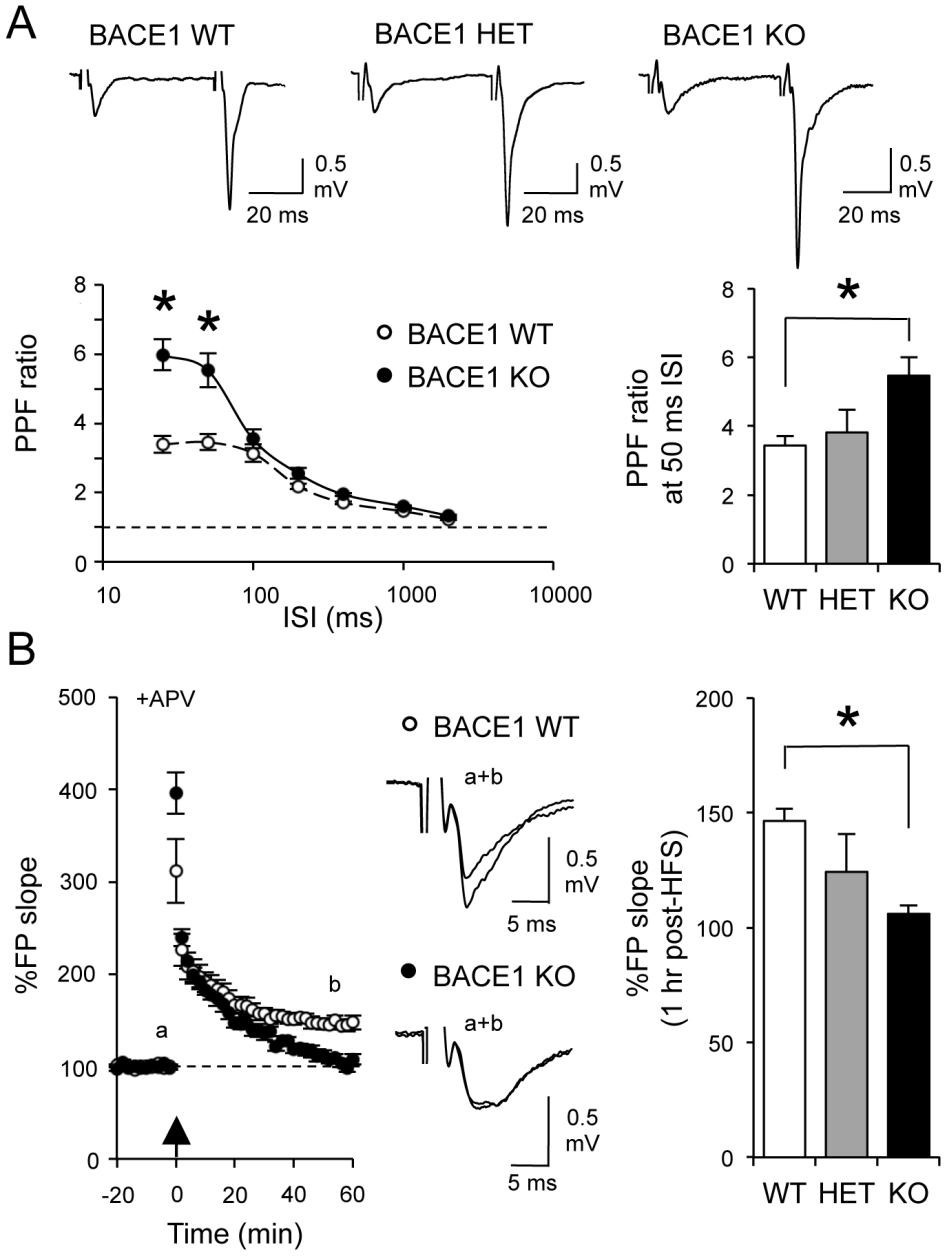
Abnormal synaptic function at MF to CA3 synapses in young BACE1 KOs. (A) Young BACE1 KOs (black circles) displayed larger PPF ratio (especially at 25 and 50 msec ISIs) compared to WTs (white circles). Top panel: Representative field potential traces following paired-pulse stimulation at 50 msec ISI. *P<0.001, two-way ANOVA; Fisher’s PLSD post hoc test P<0.001 between the two genotypes. Bottom right: BACE1 heterozygotes (HET) showed an intermediate phenotype between WT and KO at 50 msec ISI (one-way ANOVA, P<0.02; Dunnett’s multiple comparison test: *P<0.05 compared to WT). (B) Absence of mossy fiber LTP in young BACE1 KOs. Left: Summary graph plotting changes in normalized field potential against time. The arrow depicts when HFS (100 Hz, 1 sec×3) was delivered. Note that KOs (black circles) showed no LTP 60 minutes after LTP induction compared to WTs (white circles). Middle: Superimposed representative field potential traces taken from WTs and KOs at times indicated in the left panel. Right: BACE1 HETs showed an intermediate level of mfLTP (one-way ANOVA, P<0.02; Dunnett’s multiple comparison test: *P<0.05 compared to WT).

Previously, we found that in adults (3–6 months old) BACE1 KOs display severe deficits in presynaptic function at mossy fiber synapse in CA3 including an increase in paired-pulse facilitation (PPF) and an absence of mossy fiber LTP (mfLTP) [13], [15]. There may be developmental changes in synaptic function and plasticity, hence in order to use juvenile (2–3 weeks old) BACE1 KOs, essential for whole-cell recordings, we first verified with extracellular field potential recordings that these synaptic deficits do manifest at this age. We measured PPF ratio at various interstimulus intervals (ISIs), and found that like in older animals, young BACE1 KOs displayed significantly larger PPF ratios at shorter ISIs (25 ms ISI: WT = 3.4±0.24; KO = 6.0±0.44; 50 ms ISI: WT = 3.5±0.23, n = 8 slices (4 mice); KO = 5.6±0.49, n = 8 slices (4 mice); two-way ANOVA: P<0.001; Fisher’s PLSD post hoc test: P<0.001 for 25 and 50 ms ISI between WT and KO; Fig. 1A).

Next we compared mfLTP induced by high frequency stimulation (HFS: 3×100 Hz, 1 sec) from 3-week-old BACE1 WT and KO mice. Again similar to our previous data from adults, we found that young BACE1 KOs displayed a larger initial potentiation, suggesting an enhanced facilitation during HFS, yet the responses returned to baseline by 1 hour (WT: 146±5% of baseline at 1 hour post-HFS, n = 8 slices (4 mice); KO: 106±4%, n = 8 slices (4 mice); t-test: P<0.001; Fig. 1B). Consistent with a presynaptic locus of expression, LTP in WTs was accompanied by a decrease in PPF ratio measured at 50 ms ISI (baseline = 3.4±0.26, 1 hour post-HFS = 2.6±0.20, n = 8 slices (4 mice), paired t-test: P<0.001), but KOs displayed no change in PPF ratio (baseline = 5.4±0.54, 1 hour post-HFS = 5.5±0.56, n = 8 slices (4 mice), paired t-test: P = 0.54). Heterozygous (HET) mice showed an intermediate phenotype (n = 7 slices, 4 mice; Fig. 1). These results suggest that the synaptic dysfunction previously reported in adults [13], [15] is fully manifested in juvenile BACE1 KOs. Therefore, animals at this age were used in subsequent whole-cell patch-clamp recording experiments.

### Specific Dysfunction of Evoked Excitatory Responses from MF Inputs to CA3 PYRs, but not to SL-INTs, in BACE1KOs

**Figure 2:**
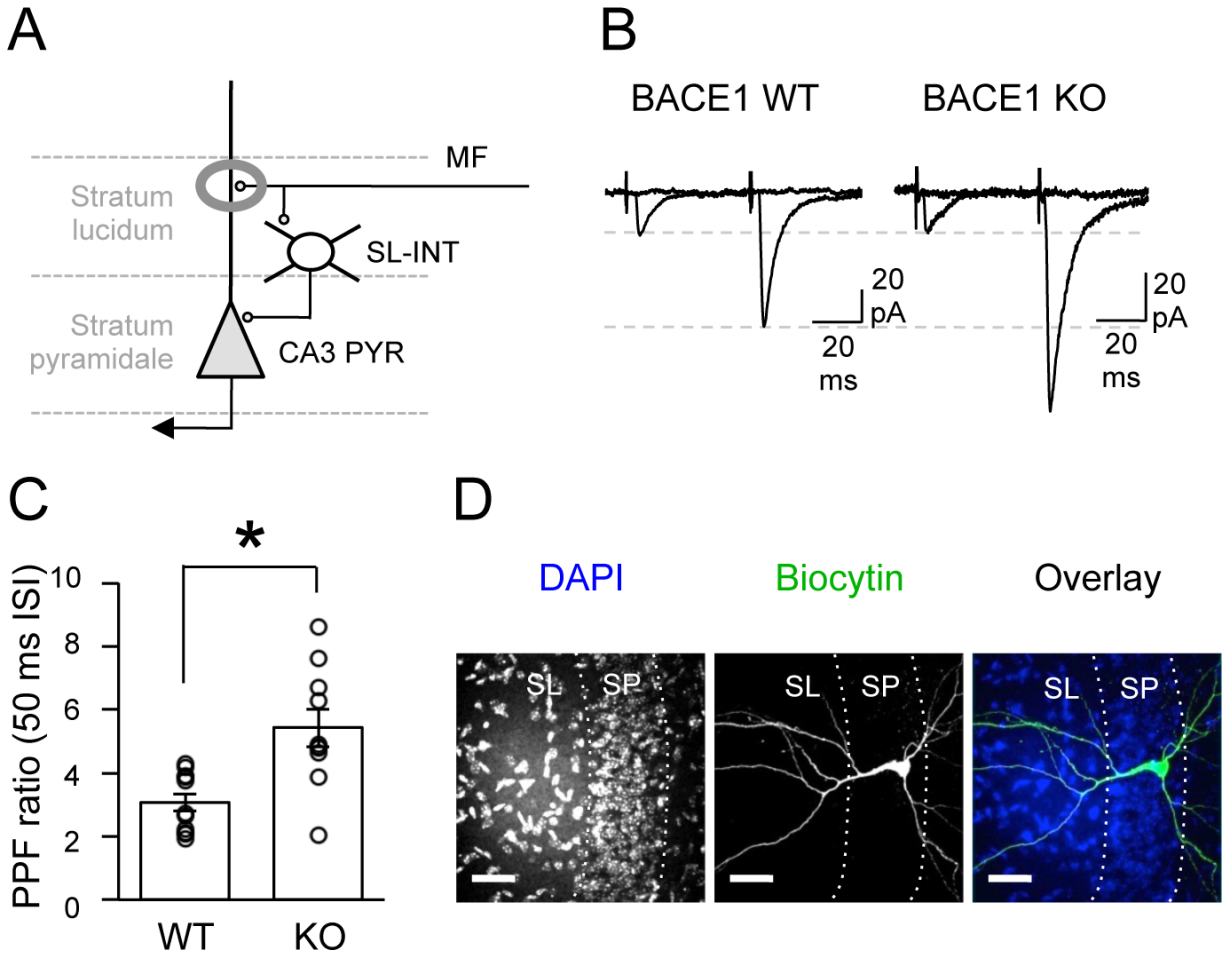
Increased paired-pulse facilitation of eEPSCs at MF inputs to CA3 PYRs in BACE1 KOs. (A) A diagram showing the CA3 circuitry. Mossy fibers (MF) form glutamatergic excitatory synapses onto CA3 pyramidal cells (PYRs) as well as onto inhibitory SL-INTs in the stratum lucidum; SL-INTs project GABAergic synapses onto CA3 PYRs. The grey circle highlights the monosynaptic excitatory synapses of MF terminals onto CA3 PYRs. (B) Representative evoked EPSC traces from CA3 PYR following paired-pulse stimulation at 50 msec ISI before and after DCG-IV application in WTs and KOs. (C) PPF ratio was significantly increased in BACE1 KOs at 50 msec ISI compared to WTs. PPF ratio of individual cells are overlayed on the bar graph as open circles. *t-test: P<0.01. (D) Immunohistochemical labeling of a representative biocytin filled CA3 PYR. Left: DAPI staining of the CA3 subfield where a biocytin filled pyramidal cell was located. Stratum pyramidale (SP) and stratum lucidum (SL) borders are marked as white dotted lines. Middle: Biocytin staining of a recorded CA3 PYR. Right: Overlay of DAPI (blue) and biocytin (green). Scale: 40 μm.

The first set of synapses we examined in the CA3 circuit was the excitatory monosynaptic MF inputs to CA3 PYRs (Fig. 2A). We stimulated dentate gyrus granule cell layer [22] and performed whole-cell patch-clamp recordings from CA3 PYRs. Monosynaptic AMPAR-mediated excitatory postsynaptic currents (eEPSCs) were isolated pharmacologically by applying D,L-APV, which is an antagonist of NMDARs, and bicuculline, which is a GABA_A_R antagonist. The latency, rise time, and decay time of the eEPSC traces were analyzed, and there were no differences in these properties between the two genotypes (t-test: P>0.6; Table 1). Consistent with our extracellular field potential recording data, BACE1 KOs displayed a significant increase in PPF ratio at 50 ms ISIs compared to WT mice (WT = 3.1±0.28, n = 11; KO = 5.4±0.61, n = 10; t-test: P<0.01; Fig. 2B,C). To verify that the responses were elicited from MF inputs, 1 μM DCG-IV was bath applied at the end of the recording, which produced a comparable reduction in basal synaptic transmission in both genotypes (WT: 20.3±3.7% of baseline, n = 11; KO: 17.6±3.6%, n = 10; Fig. 2B). We filled the recorded neurons with biocytin to verify the cell type posthoc (Fig. 2D).

**Table 1:** The properties of evoked postsynaptic currents in CA3 area of BACE1 WT and KO mice.

	Genotype	Cells	Latency (ms)	Rise time (ms)	Decay time (τ, ms)
CA3 PYRs eEPSCs	WT	n = 11	2.4±0.1	1.8±0.1	7.7±0.5
	KO	n = 10	2.5±0.1	1.9±0.2	7.9±0.6
SL-INTs eEPSCs	WT	n = 8	2.4±0.1	1.1±0.1	3.1±0.3
	KO	n = 5	2.3±0.2	1.0±0.1	3.0±0.2
CA3 PYRs eIPSCs	WT	n = 11	1.5±0.1	2.3±0.2	20.6±1.9
	KO	n = 12	1.6±0.1	2.2±0.2	20.0±1.1

**Figure 3:**
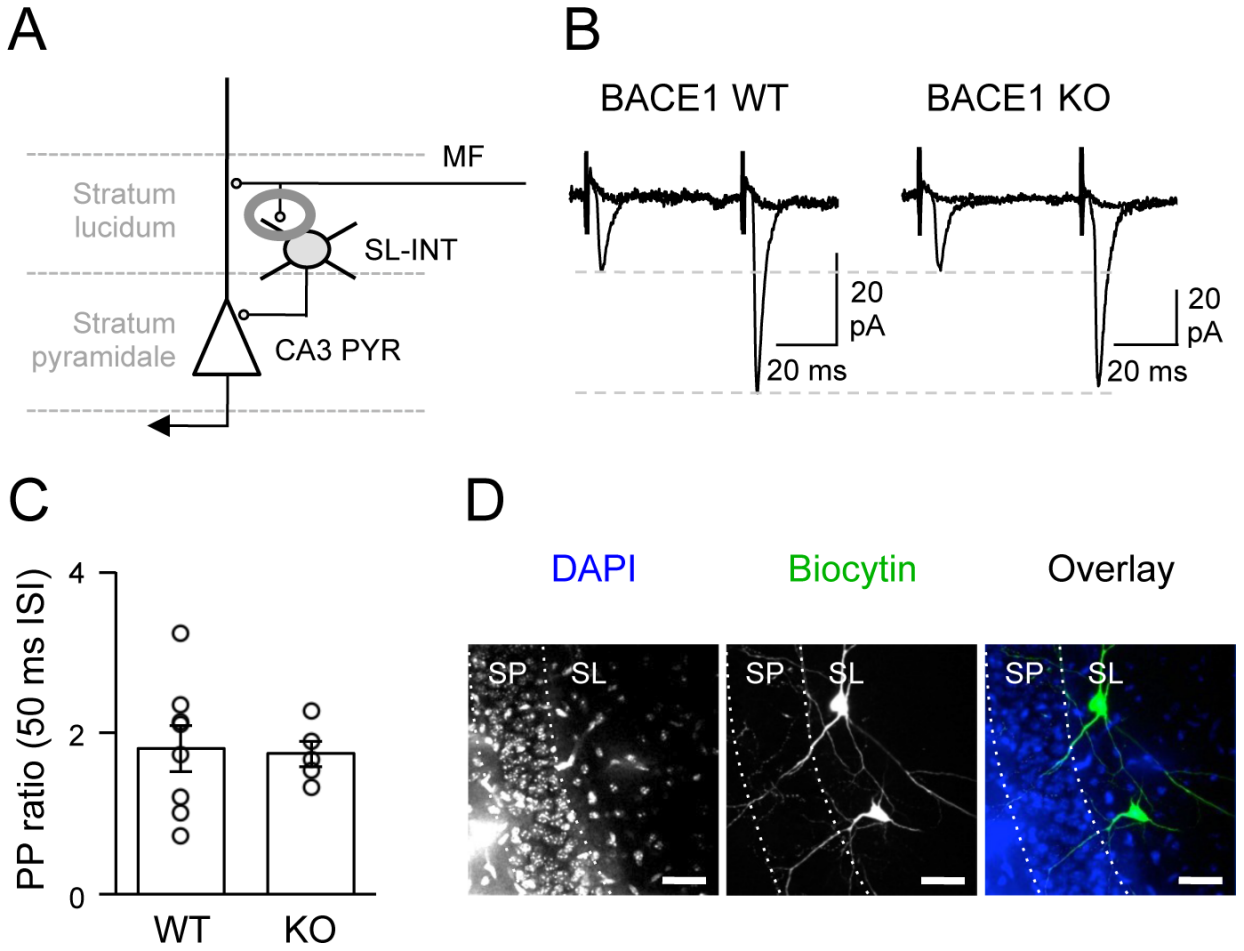
Normal synaptic function of MF inputs onto SL-INTs of BACE1 KOs. (A) A diagram highlighting the synapses of MF terminals onto inhibitory SL-INTs. (B) Representative evoked EPSC traces from SL-INTs following paired-pulse stimulation at 50 msec ISI before and after DCG-IV application in WTs and KOs. (C) Paired-pulse (PP) ratio was not changed between the two genotypes at 50 msec ISI. PP ratio of each cell is shown as open circles. (D) Immunohistochemical labeling of representative biocytin filled SL-INTs of CA3. Left: DAPI staining of the CA3 subfield where biocytin filled interneurons were located. Stratum pyramidale (SP) and stratum lucidum (SL) borders are shown (white dotted lines). Middle: Biocytin staining of two recorded SL-INTs. Right: Overlay of DAPI (blue) and biocytin (green). Scale: 40 μm.

The granule cell axons (MFs) have more than one terminal type including large complex mossy boutons, small en passant terminals, and small filopodial extensions of the mossy fiber boutons. MFs only innervate CA3 PYRs via the large complex mossy boutons, whereas small en passant and filopodial terminals preferentially target the SL-INTs [17], [23]. To assess whether the MF synapses onto SL-INTs are altered in BACE1 KOs, we isolated excitatory MF inputs on INTs in the CA3 stratum lucidum and recorded eEPSCs (Fig. 3A,B). There was no significant difference in the latency, rise time, and decay time of eEPSC traces between the two genotypes (t-test: P>0.5; Table 1). In addition, there was no change in the paired-pulse ratio between the two genotypes (WT = 1.8±0.29, n = 8; KO = 1.7±0.16, n = 5; t test: P = 0.84; Fig. 3B,C) suggesting normal synaptic function. Bath application of 1 μM DCG-IV at the end of the recording resulted in a significant reduction in basal synaptic transmission in WTs and KOs (WT: 13.4±5.5% of baseline, n = 8; KO: 12.7±6.4%, n = 5; Fig. 3B), which confirmed that we were recording MF mediated synaptic responses. To verify that the recorded cells were SL-INTs, cells were filled with biocytin and processed. Only cells located in the stratum lucidum of CA3 with interneuron morphology were included in the analyses (Fig. 3D). These data suggest that lacking BACE1 specifically alters MF inputs to CA3 PYRs, but not SL-INTs.

### Dysfunction of Inhibitory Synaptic Transmission onto CA3 PYRs in BACE1KOs

**Figure 4:**
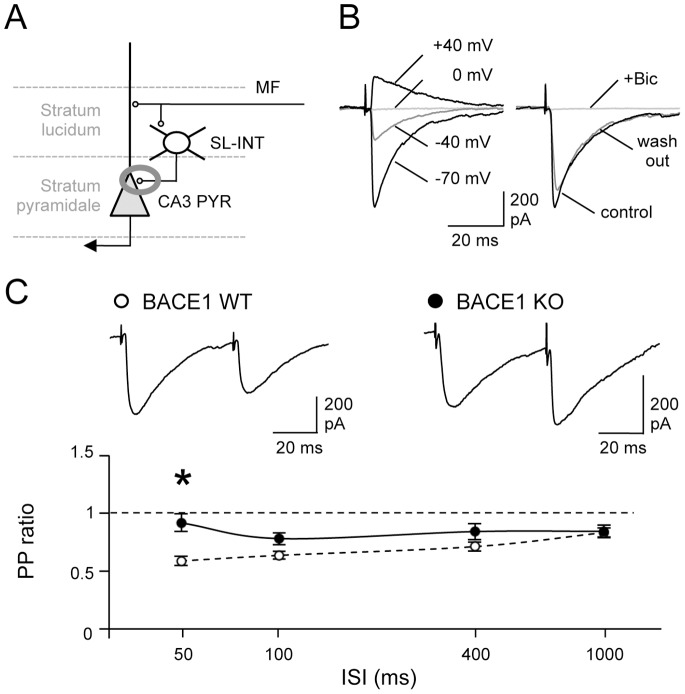
Increased paired-pulse ratio of eIPSCs from SL-INTs to CA3 PYRs in BACE1 KOs. (A) A diagram showing the inhibitory input from SL-INTs to CA3 PYRs. (B) Left: Verification that the internal solution we used reversed evoked IPSCs at 0 mV. Each evoked IPSC trace was recorded under different holding voltage as indicated. Right: The pharmacologically isolated eIPSCs were blocked by addition of 20-μM bicuculline (+Bic, gray trace), and the currents were reversible when bicuculline was washed out. (C) Paired-pulse depression was impaired in BACE1 KOs (black circles) especially at 50 msec ISI compared to WTs (white circles). Top panel: Representative evoked IPSC traces from CA3 PYRs following paired-pulse stimulation at 50 msec ISI. *P<0.001, two-way ANOVA; Fisher’s PLSD post hoc test P<0.001 between the two genotypes.

We demonstrated that BACE1 regulates excitatory synaptic transmission of MFs in a postsynaptic target specific manner. However, whether BACE1 plays a role in modulating inhibitory synapses is unknown. We therefore investigated inhibitory inputs from SL-INTs onto CA3 PYRs in BACE1 KOs (Fig. 4A) by isolating GABA_A_R-mediated IPSCs within CA3 area using AMPAR antagonist, NBQX, and NMDAR antagonist, APV. Stratum lucidum of CA3, where SL-INT cell bodies are located, were stimulated and recordings were made from CA3 PYRs. We measured evoked monosynaptic IPSCs (eIPSCs) at a negative holding potential of −70 mV using a symmetrical chloride internal solution that reverses IPSCs at 0 mV, and confirmed the isolation of IPSCs by showing a complete and reversible block of current by application of bicuculline (Fig. 4B). We did not observe significant difference in the latency, rise time and decay time of the eIPSC traces between the two genotypes (t test: P>0.4; Table 1). BACE1 WT mice showed paired-pulse depression (PPD) especially at shorter ISIs (paired-pulse ratio: 50 ms ISI = 0.59±0.04, n = 11; Fig. 4C). The paired-pulse ratio (PPR) was significantly larger in BACE1 KOs (PPR: 50 ms ISI = 0.92±0.08, n = 12; two-way ANOVA: P<0.001; Fisher’s PLSD post hoc test: P<0.001 for 50 ms ISI between WTs and KOs; Fig. 4C). To our knowledge, this is the first evidence that BACE1 regulates inhibitory synaptic transmission.

### Reduction in the Frequency of mEPSCs and mIPSCs in CA3 PYRs of BACE1 KOs

**Figure 5:**
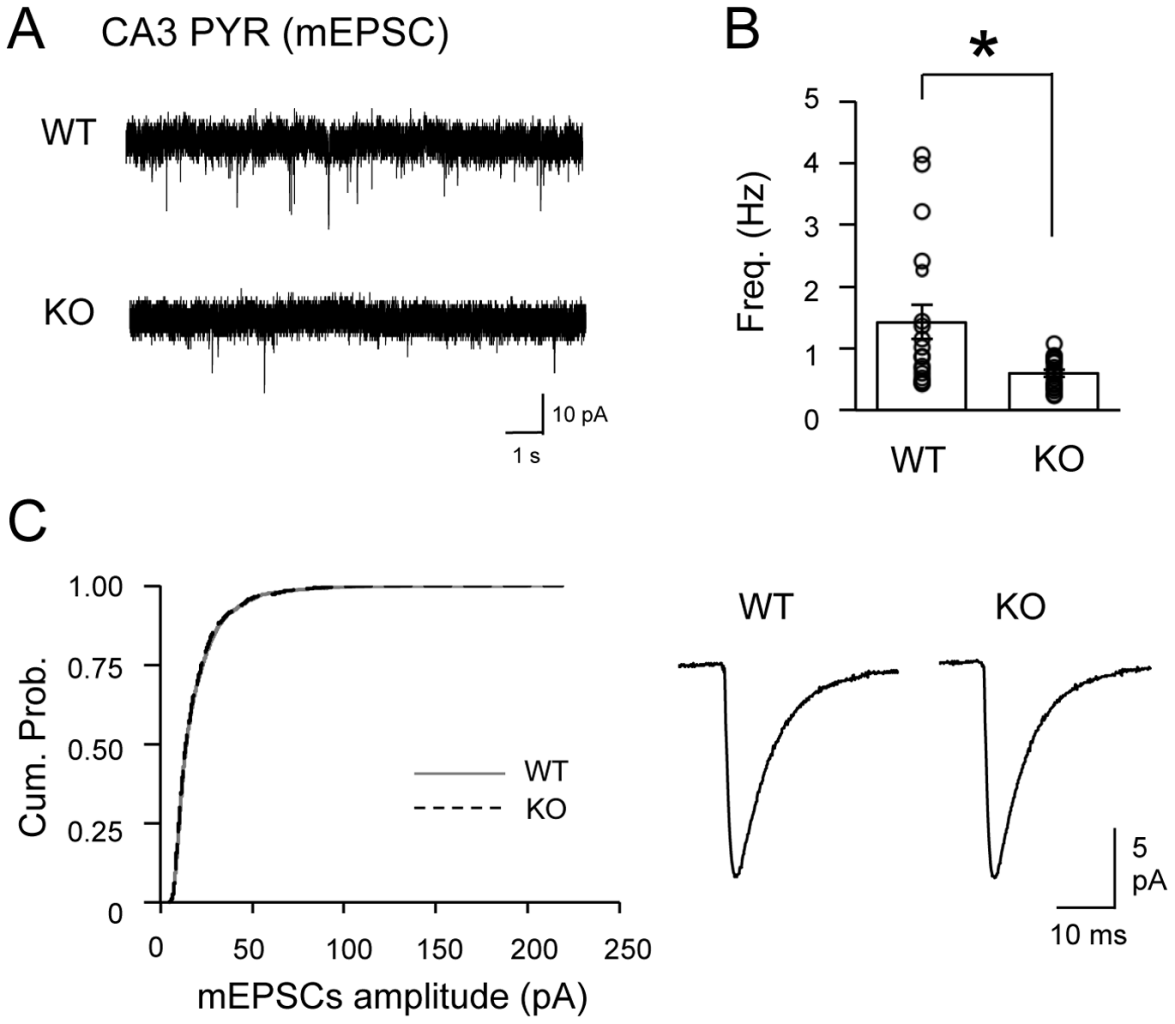
Reduced frequency of mEPSCs in CA3 PYRs of BACE1 KOs. (A) Representative mEPSC traces from CA3 PYRs in WTs and KOs. (B) BACE1 KOs showed significantly decreased mEPSCs frequency in CA3 PYRs compared to WTs. mEPSC frequency of each cell is shown as open circles. *t-test: P<0.01. (C) Amplitude of mEPSCs in CA3 PYRs was not altered in KOs. Left: The cumulative probability curve of KO mEPSC amplitudes (black dotted line) superimposed with that of WT (gray solid line) (K–S test, P>0.5). Right: Average mEPSC traces from PYRs of the two groups.

**Figure 6:**
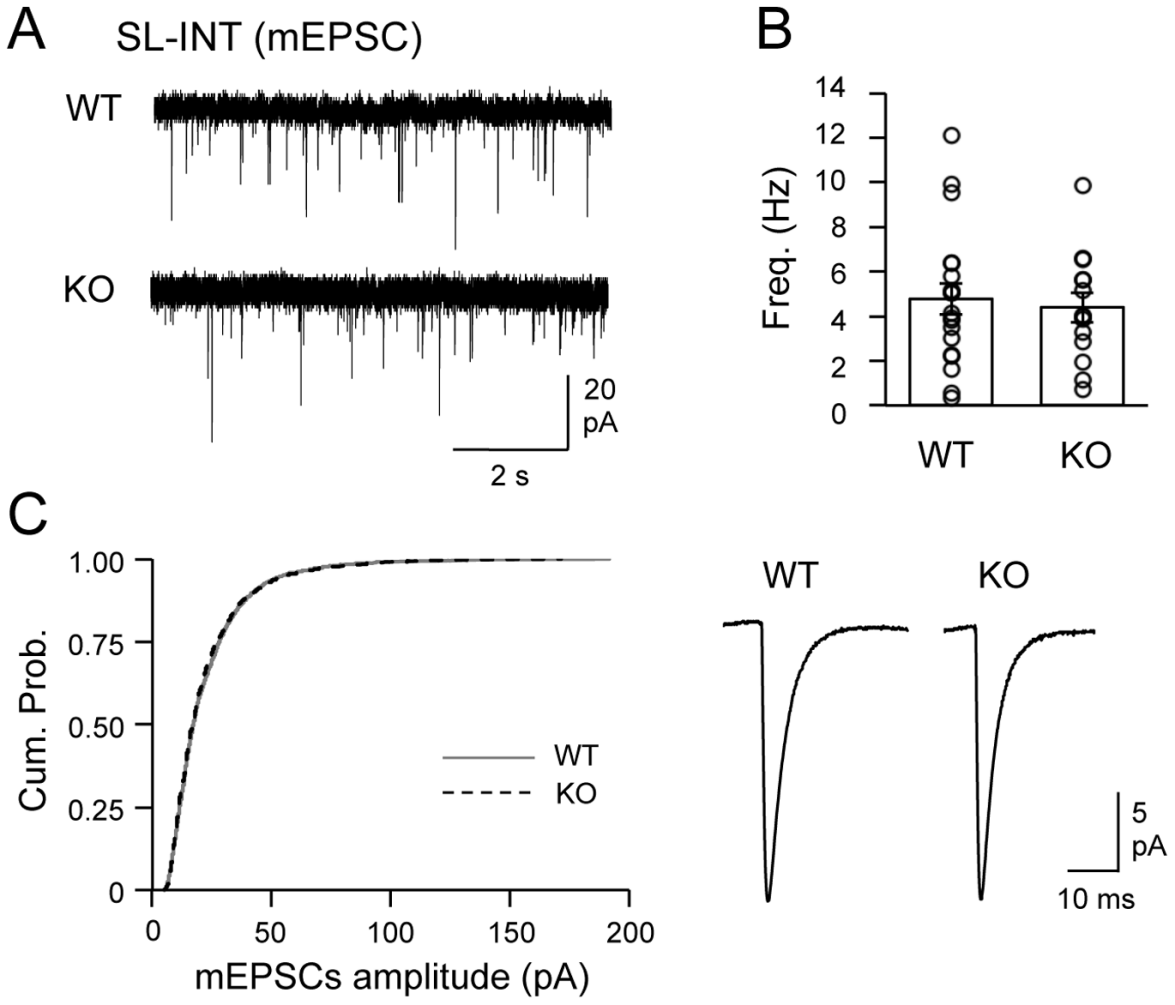
Normal mEPSCs recorded from SL-INTs of BACE1 KOs. (A) Representative mEPSC traces from SL-INTs of WTs and KOs. (B) There was no change in the frequency of mEPSCs from SL-INTs. mEPSC frequency of each cell is shown as open circles. (C) Amplitude of mEPSCs in SL-INTs was not altered in KOs. Left: The cumulative probability curve of KO mEPSC amplitudes (black dotted line) superimposed with that of WT (gray solid line) (K–S test, P = 0.2). Right: Average mEPSC traces from SL-INTs of the two groups.

Next we monitored action potential independent miniature synaptic potentials in both CA3 PYRs and SL interneurons. BACE1 KOs showed a significant reduction in the frequency of miniature excitatory postsynaptic currents (mEPSCs) recorded from CA3 PYRs (WT = 1.4±0.3 Hz, n = 19; KO = 0.6±0.1 Hz, n = 19; t test: P<0.01; Fig. 5A,B) without changes in mEPSC amplitude distribution (Kolmogorov-Smirnov test: P>0.5; Fig. 5C) nor the average mEPSC amplitude (WT = 19.4±1.0 pA, n = 19; KO = 17.5±1.4 pA, n = 19; t test: P = 0.28). In contrast, mEPSCs recorded from the SL-INTs did not change significantly in the frequency (WT = 4.8±0.7 Hz, n = 19; KO = 4.4±0.7 Hz, n = 14; t test: P = 0.69; Fig. 6A,B) or the amplitude (Kolmogorov-Smirnov test: P = 0.2; Average: WT = 22.5±2.0 pA, n = 19; KO = 22.0±2.2 pA, n = 14; t test: P = 0.88; Fig. 6C). Because mEPSCs originate from multiple synapses and input types, these results suggest that there may be a more global change in excitatory synaptic function onto CA3 PYRs without corresponding alterations in the excitatory synapses onto SL-INTs.

**Figure 7:**
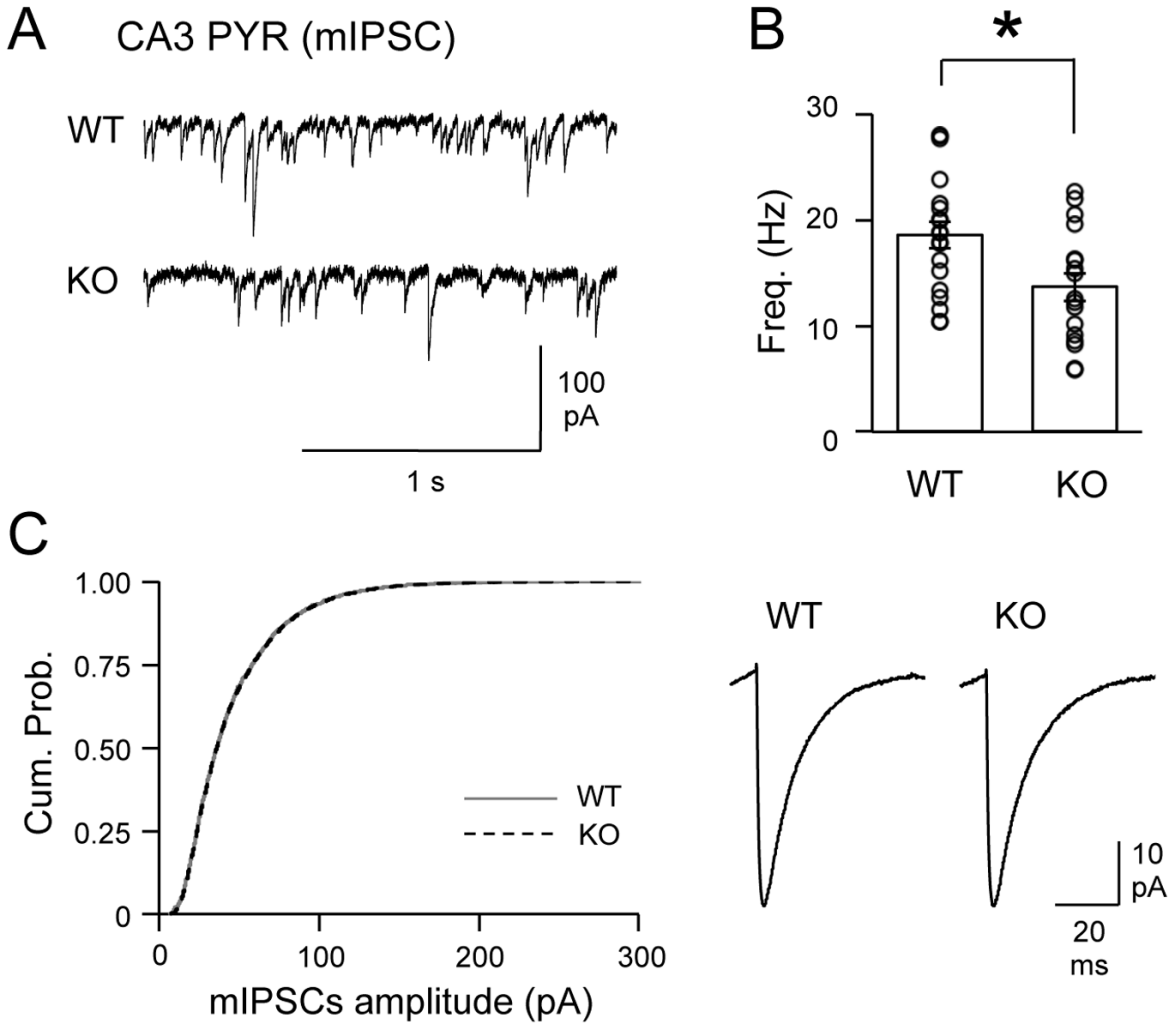
Reduced frequency of mIPSCs in CA3 PYRs of BACE1 KOs. (A) Representative mIPSC traces from CA3 PYRs in WTs and KOs. (B) BACE1 KOs showed significantly reduced mIPSC frequency in CA3 PYRs compared to WTs. Open circles are mIPSC frequency of individual cells *t test: P<0.05. (C) Amplitude of mIPSCs in CA3 PYRs was not altered in KOs. Left: The cumulative probability curve of KO mIPSC amplitudes (black dotted line) superimposed with that of WT (gray solid line) (K–S test, P>0.7). Right: Average mIPSC traces from PYRs of the two groups.

To determine whether the changes in CA3 PYRs are specific to excitatory inputs, we also compared miniature inhibitory postsynaptic currents (mIPSCs) from CA3 PYRs across the two genotypes. BACE1 KOs displayed a significant reduction in the frequency of mIPSCs (WT = 18.6±1.3 Hz, n = 19; KO = 13.7±1.3 Hz, n = 17; t test: P<0.05; Fig. 7A,B) without changes in mIPSC amplitude distribution (Kolmogorov-Smirnov test: P>0.7; Fig. 7C) nor the average mIPSC amplitude (WT = 42.2±3.2 pA, n = 19; KO = 45.7±3.6 pA, n = 17; t test: P = 0.46). Collectively, our results suggest that the function of both excitatory and inhibitory synapses is likely altered across multiple inputs onto CA3 PYRs of BACE1 KOs.

## Discussion

Our study indicates that within the CA3 stratum lucidum circuit of hippocampus, synaptic functions of both excitatory and inhibitory inputs onto CA3 PYRs are altered in the BACE1 KOs. On the other hand, there is no corresponding change in the function of excitatory synapses onto SL-INTs. Our data suggest that BACE1 can regulate synaptic transmission of both excitatory and inhibitory components within the same postsynaptic neuron. The fact that the synapses affected by BACE1 ablation were made onto CA3 PYRs reveals that this effect is likely specified by the postsynaptic target independent of the type of inputs.

Our finding that only synapses of MF inputs onto CA3 PYRs, but not MF projections onto CA3 SL-INTs, were altered in BACE1 KOs (Fig. 2–4) is consistent with the idea that even though these two sets of synapses originate from the same dentate granule cell, the functional characteristic of these two sets of synapses is quite different. Previous studies have shown that MF synapses onto CA3 PYRs display distinct high level of facilitation indicative of low release probability [20]; while MF synapses to INTs exhibit a higher probability of release and display either facilitation or depression by brief trains of stimulation [21]. Recently, it has been found that different types of AMPARs are present at the two sets of synapses. MF inputs onto CA3 PYR synapses contain only calcium-impermeable (CI) AMPARs [21]; whereas MF synapses onto SL-INTs contain both CI and calcium-permeable (CP) AMPARs [24]. Interestingly, high frequency stimulation (100 Hz), which induces LTP at MF to CA3 PYR synapses, produces NMDAR-dependent LTD at CI-AMPAR synapses [25] and NMDAR-independent LTD at CP-AMPARs containing synapses on interneurons [21]. Our results add to these differences and suggest that BACE1 function is more critical for maintaining normal synaptic transmission at the MF to CA3 PYR synapses.

Our study, for the first time, provides evidence that knocking out BACE1 not only affects excitatory inputs, but also impairs feedforward inhibition in the CA3 circuit. When interneurons and principal cells receive the same excitatory input, the inhibitory projections from interneurons onto principal cells form disynaptic feedforward inhibition [26], which shunts the excitability and tunes the firing pattern of the principal cells. Feedforward inhibition is one of the major components within neuronal circuits in many brain areas, such as hippocampal formation, visual cortex, sensory cortex, etc. [26]–[28], and plays a crucial role in circuit development, balancing excitation and inhibition to maintain neural activity [29], as well as providing precision in memory encoding [19]. In the CA3 area of hippocampus, MF terminals innervating PYRs are large with multiple release sites, whereas INTs receive small MF branches but large number of synapses. In addition, a single MF target tens of inhibitory neurons, and each inhibitory interneuron can contact hundreds of CA3 PYRs. This anatomy allows the CA3 circuit to form a high pass filter, which permits high frequency responses to pass but dampens low frequency responses by feedforward inhibition [16]. We have shown that losing BACE1 activity impairs the feedforward inhibition in CA3, which is likely to impact the function of CA3 circuit in BACE1 KOs. Thus, the previously observed behavioral deficits in BACE1 KOs [12], [30] may be in part attributed to the impairment of this inhibitory circuit.

Our finding that lacking BACE1 alters both excitatory and inhibitory synapses onto CA3 PYRs suggests that this enzyme plays a critical role in regulating synaptic inputs to these principal neurons. Changes in PPR and the frequency of mPSCs have classically been interpreted to reflect changes in presynaptic function. However, recent studies suggest that PPR changes can arise from postsynaptic alterations in AMPA receptors [31], [32], and alterations in the frequency of mPSCs can reflect changes in the number of functional synapses [33], [34]. Also, we cannot exclude the possibility that there may be changes in dendritic morphology in the BACE1 KOs, which may have affected dendritic filtering and detection of mPSCs. At this point, our results cannot distinguish between these possibilities, and further studies are needed to determine the exact locus of synaptic dysfunction. Nonetheless, our results indicate that lacking BACE1 can alter both excitatory and inhibitory synaptic function in CA3 PYRs.

The synaptic dysfunctions seen in BACE1 KOs likely arise from lacking the products of this enzyme. One potential candidate is Aβ generated by BACE1 processing of APP. Aβ has been shown to secrete from both pre- and post-synaptic neurons to alter synaptic function and plasticity [35]. Furthermore, there is accumulating evidence that Aβ may serve physiological function, and transgenic mice lacking APP cleaving enzymes show specific synaptic dysfunctions (reviewed in [14]). Synaptic deficits seen in BACE1 KOs may also be due to other functions of BACE1 independent of its enzymatic activity for Aβ production. Recently Chen et al. reported that the transmembrane domain of BACE1 interacts with adenylyl cyclase, which in turn down regulates cAMP/PKA/CREB pathway. They also found that the levels of synaptophysin and PSD-95 were decreased in the brain of BACE1 KO mice indicating both pre- and post-synaptic changes [36]. It is also possible that the synaptic dysfunctions found in BACE1 KOs may be from abnormal processing of neuregulin-1 (NRG1), another substrate of BACE1 besides APP [37], [38], which affects presynaptic release by regulating the surface expression of presynaptic Ca^2+^-permeable α7-nAChRs [39]–[41]. Presynaptic nAChRs can increase glutamate release [42]–[44], likely via the α7-nAChRs [45]. Indeed, by activating α7-nAChRs, we were able to rescue the presynaptic deficits seen from extracellular field recording at MF to CA3 pathway in BACE1 KOs by recruiting calcium-induced calcium release (CICR) [15]. Whether activation of α7-nAChRs can restore synaptic deficits seen at the inhibitory synapses on PYRs is not clear, and needs further investigation. Recent work showed that NRG1 is involved in regulating excitatory synapses onto GABAergic interneurons [46]. Therefore, it is of interest that we did not observe changes in excitatory synaptic transmission on SL-interneurons in BACE1 KOs, and that the effects are specific to excitatory synapses on CA3 pyramidal neurons.

Even though it is possible that other sources of excitatory and inhibitory inputs besides the ones we examined are altered in BACE1 KOs, our results clearly show that both excitation and inhibition onto CA3 PYRs are changed in the absence of BACE1. It is pertinent to note that recent studies indicate that dysfunction in CA3 contributes to learning deficits in aging [47], [48] and Rett syndrome [49]. The direction of changes we observed for both excitatory and inhibitory components of CA3 PYRs were the same (i.e. decrease in mPSC frequency and increase in PPR), which may reflect a compensatory mechanism to keep the E/I balance. In any case, these changes likely impact synaptic function in the CA3 area of hippocampus as seen by an abolishment of mfLTP [13], [15]. The drastic phenotype seen for mfLTP in the BACE1 KOs contrasts the minimal deficits in LTP at the Schaffer collateral inputs to CA1 [12]. Studies have reported that partial ablation of BACE1 recovers synaptic plasticity deficits seen in CA1 region of transgenic models of AD [50], [51]. Our observation of an intermediate phenotype with BACE1 HETs suggests that BACE1 is critical role for the normal physiology of CA3 neurons and warrants examination of CA3 synaptic plasticity in AD transgenic models. One possible confounds of our present study, which is common to all studies utilizing gene knockout or transgenic mouse models, is that it is nearly impossible to distinguish direct effect of lacking BACE1 from compensatory changes that are secondary to lacking the enzyme. However, because compensatory changes are also expected for long term BACE1 inhibition, we believe our results would be relevant for any clinical interventions targeting BACE1. In sum, the current study reveals BACE1 function at a circuit level, and may provide useful mechanistic information to circumvent the negative side effects caused by BACE1 inhibition for AD treatment.
